# Severity of Tinnitus Distress Negatively Impacts Quality of Life in Patients With Vestibular Schwannoma and Mimics Primary Tinnitus

**DOI:** 10.3389/fneur.2019.00389

**Published:** 2019-04-24

**Authors:** Takashi Kojima, Naoki Oishi, Takanori Nishiyama, Kaoru Ogawa

**Affiliations:** Department of Otolaryngology, Head and Neck Surgery, Keio University School of Medicine, Tokyo, Japan

**Keywords:** acoustic neuroma, tinnitus, hearing loss, vertigo, anxiety, quality of life, prospective studies, conservative treatment

## Abstract

**Objective:** Quality of life (QoL) and subjective symptoms are predominantly used to evaluate treatment outcome of patients with vestibular schwannoma (VS). However, for patients undergoing conservative treatment—the most frequently used intervention—the association between QoL and subjective symptoms is unclear. Moreover, it is unknown whether VS-related tinnitus could be associated with the audiological and psychological status of the patient. Our overall aim is to provide objective evidence of this association to better guide treatment of VS.

**Methods:** In a prospective study, we analyzed factors that influence VS-related tinnitus and QoL in 72 patients receiving conservative management of unilateral sporadic VS. This was done through questionnaires that assessed QoL, anxiety, depression, and audiological examinations. We used the SF-36 Short Form to assess QoL; the Tinnitus Handicap Inventory, Dizziness Handicap Inventory, Facial Clinimetric Evaluation Scale, Visual Analog Scale for hearing impairment to assess symptoms subjectively; and pure tone audiometry, the speech discrimination for hearing measurements. For psychological status, we used the Hospital Anxiety and Depression Scale. For analyses, we used Pearson correlation analysis and multiple regression between variables and QoL.

**Results:** Correlation and regression analyses revealed that the severity of tinnitus distress had the largest negative impact on QoL in all domains of SF-36. The severity of tinnitus was significantly associated with subjective hearing impairment and the degree of depression and anxiety. Hearing thresholds had no statistical association with severity of tinnitus.

**Conclusions:** To our knowledge, this is the first study to investigate VS-related tinnitus with respect to both patients' hearing status and psychological condition. Our results suggest that tinnitus distress strongly affects VS patients' QoL and that its characteristics are similar to primary tinnitus. An intervention for VS-related tinnitus, therefore, should assess to what extent tinnitus bothers patients, and it should reduce any unpleasant emotions that may exacerbate symptoms. This approach should improve their QoL.

## Introduction

Vestibular schwannoma (VS)—or acoustic neuroma—is a benign, slow-growing tumor of myelin-forming cells of the vestibulo-cochlear nerve. It usually causes progressive firstly unilateral hearing loss, then tinnitus or/and vertigo appear next, and more progressive cases shows facial paresis. An actively growing VS is disabling if it presses against nearby brain structures. Conservative management using a wait-and-scan protocol is adopted for more than half of VS patients (1). The remaining patients receive active treatment, which includes radiotherapy (gamma knife or cyber knife) and microsurgery for tumor excision (1, 2). Regardless of whether conservative management or active treatment is selected, quality of life (QoL) is decreased and anxiety is elevated (3, 4).

In recent years, health-related QoL has been the primary measure to assess patient-associated outcomes in patients with VS (1, 3). The Medical Outcomes Study Short Form Health Survey (SF-36) is also widely accepted and used for evaluating overall QoL in patients with VS (3). Several studies have compared the outcome of microsurgery and radiotherapy, generally finding that QoL is worse after microsurgery (5, 6). Other studies indicate that patients experience better QoL after conservative management than with active treatment (7, 8). Even though conservative management is the most frequently conducted intervention for VS patients, there is a lack of studies that focus on how conservative management affects the QoL of these patients.

Most VS research studies use QoL to assess the severity of subjective symptoms, because audiological examinations, such as assessing hearing thresholds or tumor size, have a weaker statistical association (3). The SF-36 has been the most frequently used tool to assess patients with VS, but it has not been used for assessing disease-related symptoms. One instrument that has been developed for measuring disease-specific QoL is the Penn Acoustic Neuroma Quality-of-Life Scale (PANQOL) (9). This questionnaire implies a unique property for assessing disease-specific conditions; it comprises eight domains (hearing, balance, facial, pain, anxiety, vitality, general, and total) (9). However, tinnitus was excluded as one of the test items, even though 60–70% of patients with VS experience tinnitus (2, 10). Perhaps tinnitus was excluded because this prominent symptom has not received sufficient attention in VS patients. Indeed, only a few studies have analyzed the relationship between generic QoL and severity of VS-related symptoms, like tinnitus, using validated questionnaires (11, 12).

Generally, distress suffering from primary spontaneous chronic tinnitus is a major factor that affects QoL (11, 13). However, for patients with VS, neither the existence (10) nor the duration (8) of tinnitus has been shown to be statistically associated with overall QoL. For clinical practice, an assessment with a validated questionnaire is more meaningful for evaluating characteristics of tinnitus (14). Many factors contribute to primary tinnitus, and past studies indicate that both hearing loss (15) and the psychological condition of patients can affect tinnitus severity (16). However, to the best of our knowledge, no study has investigated the contribution of both auditory and psychological factors to VS-related tinnitus and how these factors affect QoL.

Our research aimed to determine which factors influence QoL and the severity of VS-related symptoms. We investigated characteristics of tinnitus in patients with VS by analyzing both audiological measurements and subjective auditory factors as well as patients' psychological condition. We hypothesize that VS-related tinnitus is negatively correlated with QoL: the influence of VS-related tinnitus on QoL may have been underestimated, and this is related to an underappreciation of the characteristics of VS. The results of this study will provide a better understanding of what impact the severity of subjective symptoms has on QoL in VS patients, and will help clinicians determine an appropriate treatment strategy for tinnitus in patients undergoing conservative management of VS.

## Methods

### Setting and Clinical Assessment of Patients

We conducted a prospective observational study in patients (*n* = 74) undergoing conservative management of VS between December 2016 and December 2017 at VS speciality clinic, where only a patient who had already been diagnosed as VS could enroll. We presumed the VS population based on the reported incidence in Denmark (19 out of every one million individuals per year) (17); therefore, 126 million of Japanese population estimated approximately 2400 VS population. Then, we calculated the required sample size as sixty-six (confidence level was 90% and the margin of error was 10%). The definitive diagnosis had been performed by MRI to exclude differential diagnoses before enrolment including unilateral sensorineural hearing loss, Ménière's disease, or Ramsay-Hunt syndrome. Inclusion criteria for conservative management were minimal symptoms, no or slowly growing tumor, no sign of pressure on brainstem or cerebellum, older than 60 years old, physician judged that patient is not suited for invasive treatment, or patient preference for conservative treatment. Patients were excluded if they had neurofibromatosis type 2, previous or planned microsurgical treatment, or radiotherapy. Due to the property of our cross-sectional hospital, it was difficult to grasp the date of their diagnosis accurately. We measured the disease duration, therefore, between the first visit to VS speciality clinic and the day we took the questionnaires.

Tumor size and auditory function were assessed using the guidelines of the American Academy of Otolaryngology-Head and Neck Surgery (AOHNS) (18). All patients underwent annual periodic MRI within 4 years from the first visit. After 4 years of our management, MRI was undertaken once in 2 years. To estimate tumor size, the largest size on the axial MRI was selected. Two linear measurements made at the extracanalicular portion: the first diameter in the direction parallel to the petrous ridge and the second in the orientation perpendicular to the first diameter. We calculated as the square root of the product of these two diameters. Then we reported the size adding the diameter on intracanalicular diameter to the extracanalicular portion estimated on the same slice on MRI. If the tumor limited to the internal auditory canal, we only measured the intracanalicular diameter. Pure-tone audiometry (PTA) was measured once per year and was summarized as the average of the hearing thresholds assessed at 0.5, 1, 2, and 3 kHz. A hearing level classification was based on the participants' PTA results and speech discrimination score (SDS). Class A participants had a PTA of ≤30 dB and SDS of ≥70%; class B participants had a PTA of ≤50 dB and SDS of ≥50%; class C participants had a PTA of >50 dB and SDS of ≥50%; and class D participants had any PTA threshold and SDS of less than <50%. Classes A and B indicate that hearing levels in the ear on the affected side are serviceable, whereas classes C and D indicate that hearing levels in the ear on the affected side are non-serviceable.

### Questionnaires to Assess Symptoms and QoL

Participants completed six questionnaires at our clinic that assessed subjectively experienced symptoms and QoL. The first questionnaire, the SF-36, is a widely used instrument to assess QoL (19, 20). It has been translated into Japanese and was validated (19–21). The SF-36 evaluates eight health-related domains, as perceived by the participant (19, 20). These eight are (1) limitations in physical activities because of health problems (indicated here as PF); (2) limitations in social activities because of physical or emotional problems (SF); (3) limitations in role activities because of physical health problems (RP); (4) bodily pain (BP); (5) general mental health in terms of psychological distress and well-being (MH); (6) limitations in role activities because of emotional problems (RE); (7) vitality in terms of energy and fatigue (VT); and (8) general health perceptions (GH).

We calculated norm-based scores for the SF-36, which have a mean of 50 and a standard deviation of 10. This method involves the standardization of each transformed score by comparing participants' scores with those in the normal population (19, 20). The lower the score, the more disability; the higher the score, the less disability. If our participants had a mean domain score of <50, then their QoL was worse than average; and if their score was >50, then their QoL was better than the average score in the normal population. A score of 3 or more points above or below 50 represented a significantly better or worse QoL, respectively, than the QoL in the normal population.

Three questionnaires were used to assess VS-related symptoms: the Tinnitus Handicap Inventory (THI) (22), which assesses subjective severity of tinnitus distress; the Dizziness Handicap Inventory (DHI) (23), which measures the severity of dizziness and vertigo; and the Facial Clinimetric Evaluation Scale (FaCE) scale (24), which measures the severity of facial paresis. THI (25), DHI (26), and FaCE (27) were validly translated into Japanese. The THI and DHI are both scored on a scale of 0 to 100, with 0 representing no handicap and 100 representing maximum handicap. The FaCE is scored on a scale of 0 to 75, with 75 representing no handicap and 0 representing maximum handicap.

We classified the severity of the patients' tinnitus into four groups based on their THI scores: a score of 17 or below reflected negligible tinnitus, a score of 18 to 36 reflected mild tinnitus, a score of 37 to 56 reflected moderate tinnitus, and a score of 57 or above reflected severe tinnitus. If they scored higher than 17 on the THI indicating more than a mild handicap, we designated patients as having “bothersome tinnitus.”

To assess the participants' subjective hearing status, we developed a 100-mm Visual Analog Scale (VAS) that focused on unilateral hearing impairment. We developed the VAS because, at the time of our study, there was only a validated Japanese translation of the Hearing Handicap Inventory for adults (HHI-A) (28). The Hearing Handicap Inventory for elderly (HHI-E) (29) was not available. The VAS comprised six questions (translated into English here): (1) “How much does your hearing impairment bother you?” (2) “How much does your hearing loss hinder you in every-day life?” (3) “How difficult is it for you to communicate in one-to-one situations?” (4) “How difficult is it for you to talk in group conversations?” (5) “How difficult is it for you to talk when there is surrounding noise?” (6) “How difficult is it for you to perceive from where a sound originates?” Each was scored on a 1-to-100 scale. An overall VAS score was calculated by averaging the summed scores of each question. A VAS score of 0 represented no handicap, and a score of 100 represented maximum handicap. To validate VAS, we settled an expert committee which was composed of four otolaryngologists specialized in otology and audiology. The committee reviewed all items and confirmed consensus that all item can reflect unilateral hearing impairment. Then we calculated Cronbach's alpha to confirm an internal validity using the data from this study. We confirmed a reliability value of 0.958 on this questionnaire, and alpha on each item was within 0.8 to 0.958 (item 1, 0.949; item 2, 0.949; item 3, 0.957; item 4, 0.943; item 5, 0.946; item 6, 0.954). VAS and each item on this questionnaire showed adequate validity and reliability.

To assess the psychological status of the participants with conservatively managed VS, we asked them to complete the Hospital Anxiety and Depression Scale (HADS) (30), which validly translated in Japanese (31). The HADS comprises 14 items divided into two subscales: Anxiety (HADS-A) and Depression (HADS-D). Each item is scored on a scale of 0–3; therefore, the total score for each subscale can range from 0 to 21, with 21 representing the strongest symptoms of anxiety or depression.

### Analyses

First, we assessed the relationship between QoL and severity of subjective symptoms using continuous variables. We performed Pearson's correlation analysis between each SF-36 domain score and THI, DHI, FaCE, VAS scores, and age as a variable to evaluate the impact of aging on the other variables (e.g., presbycusis, declination of general health, etc.). For multiplicity adjustment of 40 items of correlation analysis, we adopted a false discovery rate (FDR) adjustment α = 0.05(32). Then multiple linear regression analysis was performed which factors influence QoL. We set a score of each domain of SF-36 as a dependent variable, and age, THI, DHI, FaCE and VAS scores as independent variables. This meant we performed eight different multiple regression analyses reflecting each SF-36 domain. The standardized coefficient beta (β) was estimated as a coefficient parameter. Second, we determined which factors influence the severity of tinnitus distress. In this analysis, we used the following as continuous variables: PTA, SDS, and VAS for assessing auditory function; and HADS-A and HADS-D for assessing psychological status. Pearson's correlation analysis between THI and variables. Then multiple linear regression analysis was done to determine which parameter predicted THI. The significance threshold was set at < 0.05. Statistical analysis was performed with SPSS Statistics software version 24 (IBM Corp. Released 2016. IBM SPSS Statistics for Windows, Version 24.0. Armonk, NY: IBM Corp.). We used a standard deviation of the mean (SD) in descriptive statistics.

### Ethical Approval

Details of this clinical research study were displayed in a consultation room, and oral consent was collected from all participants. According to the Japan Ethical Guidelines for Medical and Health Research Involving Human Subjects, obtaining informed consent for observational studies is not required. We notified the research subjects, or made public, information concerning the research, including the purpose of collecting and using the research information. We also informed the participants that they could refuse participation at any time or could request that their data be removed from the study after commencement. This information was also documented in each patients' medical chart. The study was conducted in accordance with the Declaration of Helsinki, and approved by the ethics committee of the Keio University School of Medicine (JPRN-UMIN000008901). All patients consented that their data could also be used for future studies. Data were anonymized at the time of collection.

## Results

### Patients With VS

Of 74 patients who received conservative management, 72 (97.3%) agreed to participate in our surveillance study. The questionnaires were the SF-36, THI, DHI, FaCE, subjective hearing status VAS, and the HADS. Thus, these 72 participants (33 males; 39 females) contributed the data for analysis.

**Figure 1:**
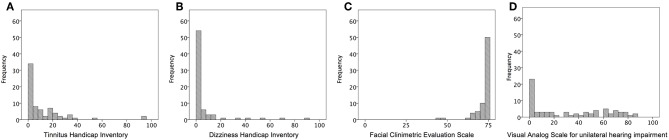
Representative histograms of the distribution of the questionnaires for severity of VS-related symptoms. **(A)** scores of Tinnitus Handicap Inventory; **(B)** scores of Dizziness Handicap Inventory; **(C)** scores of Facial Clinimetric Evaluation Scale; **(D)** Visual Analog Scale for unilateral hearing impairment.

Overall patient characteristics are shown in Table 1. The mean age was 60.0 ± 12.9 years (range, 26–84) at their assessment visit. The average tumor size was 12.8 ± 7.3 mm along the largest dimension. The mean elapsed time from the first visit of our hospital to assessment visit was 52.2 ± 51.2 months (range, 1 month to 18 years). On the affected side, an average hearing threshold was 43.6 ± 28.1 decibels and speech discrimination was 72.4%. On the contralateral side, the average hearing threshold was 18.9 ± 12.1 decibels, and speech discrimination was 92.6 ± 6.5%. According to AAOHNS classification, twenty-eight (38.9%) participants had a class A hearing level, 16 (22.2%) had a class B hearing level, 15 (20.8%) had a class C hearing level, and 13 (18.1%) had a class D hearing level. According to these criteria, 44 (61%) of the participants had a serviceable hearing, whereas the remaining 28 (39%) had a non-serviceable ear on the affected side. Figure 1 showed the distribution of a score of self-writing questionnaires. A score of THI, DHI, and VAS skewed to mild score possibly due to population enrolling conservative management. The median scores were as follows: THI was 4 (range, 0–94); DHI was 0 (range, 0–88); FaCE was 75 (range, 43–75); and VAS was 25 (range, 0–85).

**Table 1:** Overall patient characteristics in this study.

Gender (male/female)	33/39
Laterality (right/left)	35/37
	**Mean ± SD; min to max**
Age (y/o)	60.0 ± 12.9; 26 to 84
Size (mm)	12.8 ± 7.3; 2.0 to 31.4
duration (month)	52.2 ± 51.2; 1 to 216
Affected side: hearing threshold (dB)	43.6 ± 28.1; 3.8 to 110
Affected side: speech discrimination (%)	72.4 ± 33.3; 0 to 100
Contralateral: hearing threshold (dB)	18.9 ± 12.1; 3.8 to 63.8
Contralateral: speech discrimination (%)	92.6 ± 6.5; 60 to 100

### Relationship Between QoL and Subjective Symptoms of VS Patients

**Figure 2:**
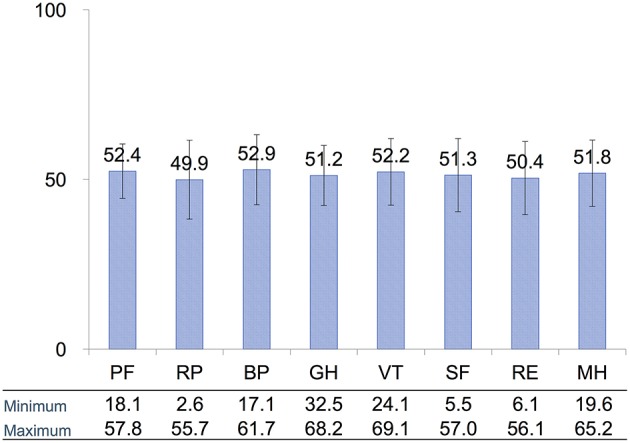
Distribution of average scores for each of the eight SF-36 domains assessing QoL. All domain scores were within the norm-based average score for each domain. PF, physical functioning; RP, role physical; BP, bodily pain; GH, general health; VT, vitality; SF, social functioning; RE, role emotional; MH, mental health. Error bars are SD.

We first determined whether QoL, as assessed with a widely used audiological examinations, is related to subjective symptoms of tinnitus in patients receiving conservative management of VS. Figure 2 presents the average score for each of the eight SF-36 domains. The domains relate to physical functioning, bodily pain, general health, vitality, social role functioning, emotional role functioning, and mental health (19, 20) (See Methods for full description). Although the average scores for each domain were within the range of norm-based average scores, some patients had scored quite low on some of the domains.

The results of correlation analysis examining the bivariate associations between patients' scores on the domains of the SF-36 and variables, which included age and their scores on the self-reports of tinnitus (THI), dizziness (DHI), facial paresis (FaCE), and subjective hearing status (VAS), are shown in Table 2. No statistical relationship between generic QoL and age in our participants after FDR adjustment. The scores for all SF-36 domains were significantly, but negatively, correlated to scores of tinnitus severity even after adjustment. This means that, as the severity of tinnitus increased, the overall QoL of the patients decreased. The QoL picture was different for participants' dizziness. Of the eight SF-36 domains, only PF, RP, SF, and RE were negatively correlated with dizziness (DHI scores), and GH, VT, and MH were negatively correlated with hearing impairment (VAS scores).

**Table 2:** Correlation results (Pearson correlation coefficients) from the assessment of the relationship between SF-36 domains and age and subjective symptoms.

	**Age**	**THI**	**DHI**	**FaCE**	**VAS**
	***r* (*p*)**	***r* (*p*)**	***r* (*p*)**	***r* (*p*)**	***r* (*p*)**
PF	−0.02 (0.869)	**−0.497 (0.000)**	**−0.459 (0.000)**	0.238 (0.044)	−0.185 (0.120)
RP	0.153 (0.199)	**−0.585 (0.000)**	**−0.386 (0.001)**	0.208 (0.079)	−0.132 (0.270)
BP	0.131 (0.272)	**−0.361 (0.002)**	−0.273 (0.020)	0.155 (0.192)	−0.177 (0.137)
GH	0.151 (0.204)	**−0.475 (0.000)**	−0.187 (0.115)	0.179 (0.134)	**−0.338 (0.004)**
VT	0.257 (0.029)	**−0.525 (0.000)**	−0.216 (0.069)	0.104 (0.386)	**−0.320 (0.006)**
SF	0.153 (0.200)	**−0.633 (0.000)**	**−0.392 (0.001)**	0.207 (0.080)	−0.264 (0.025)
RE	0.236 (0.046)	**−0.587 (0.000)**	**−0.363 (0.002)**	0.200 (0.091)	−0.248 (0.035)
MH	0.251 (0.034)	**−0.455 (0.000)**	−0.117 (0.329)	0.136 (0.254)	**−0.341 (0.003)**

*Pearson's correlation analysis was performed. Bolded measures indicate measures that remained significant after False Discovery Rate adjustment. THI, Tinnitus Handicap Inventory; DHI, Dizziness Handicap Inventory; FaCE; Facial Clinimetric Evaluation Scale; VAS, Visual Analog Scale for hearing impairment; SF-36, abbreviations are the same as in Figure 2*.

Multiple regression analysis revealed that THI was significantly related to all SF-36 domains, indicating that increasing severity of tinnitus significantly decreased the QoL of our participants (Table 3). Dizziness (DHI) and hearing impairment (VAS), on the other hand, had significant but less impact on QoL. Aging affected only vitality and emotional and mental health aspects of QoL but not other aspects contributing to QoL. Multiple regression analysis also revealed no relationship between FaCE and SF-36 scores, indicating that facial paresis had little or no influence on QoL, as measured by the SF-36. Taken together, our results show that tinnitus severity (THI) is the strongest predictor of whether QoL will be affected in conservatively managed VS patients.

**Table 3:** Multiple regression analysis of QoL and the variables age and severity of subjective symptoms.

**SF-36 DOMAIN**	**PF**	**RP**	**BP**	**GH**	**VT**	**SF**	**RE**	**MH**
Age					0.242^*^		0.196^*^	0.257^*^
THI	−0.369^*^	−0.585^**^	−0.361^*^	−0.475^**^	−0.445^**^	−0.633^**^	−0.576^**^	−0.362^*^
DHI	−0.313^*^							
FaCE								
VAS					−0.225^*^			−0.265^*^
R^2^	0.328	0.342	0.13	0.226	0.367	0.4	0.383	0.321

We set a score of each domain of SF-36 as a dependent variable, and age, THI, DHI, FaCE and VAS scores as independent variables. THI was a statistical predictor of QoL for all domains of the SF-36. Standardized beta (β) coefficients are shown.

* p < 0.05,

** p < 0.01. Same conventions as in Table 2.

### Analyses of Tinnitus Characteristics in Patients With VS

**Figure 3:**
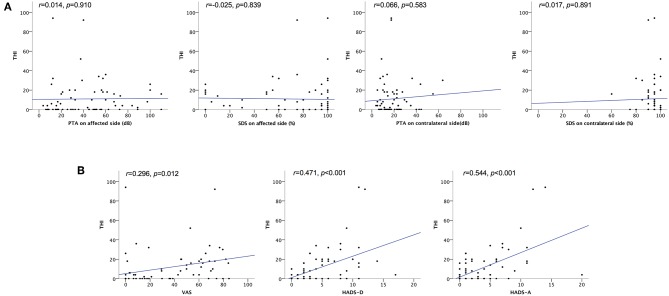
Scatter plots showing correlation analyses of the relationship between tinnitus severity and hearing impairment, depression, and anxiety. **(A)** Correlation between THI scores and hearing threshold. No statistical correlation was found between tinnitus severity (THI scores) and auditory thresholds. **(B)** Correlation between THI scores and VAS, HADS-D, and HADS-A scores. Pearson's correlation analysis revealed a significant correlation between THI scores and VAS, HADS-D, and HADS-A scores. HADS-A, Hospital Anxiety and Depression Scale Anxiety score; HADS-D, Hospital Anxiety and Depression Scale Depression score; PTA, pure tone audiometry; SDS, speech discrimination; THI, Tinnitus Handicap Inventory; VAS, Visual Analog Scale for unilateral hearing impairment; *r*, Pearson's r.

The severity of tinnitus was measured using the THI. Of the 71 patients who answered THI adequately, 53 (74.6%) had negligible tinnitus, 15 (21.1%) had mild tinnitus, 1 (1.4%) had moderate tinnitus, and 2 (2.8%) had severe tinnitus. Thus, 18 patients (25.0%) had bothersome tinnitus. Figure 3 presents bivariate scatter plots showing correlation analysis results for tinnitus distress (THI), the hearing threshold (PTA, SDS), hearing impairment (VAS), anxiety (HADS-A), and depression (HADS-D). Tinnitus distress was significantly correlated with subjective hearing impairment (VAS, *r* = 0.296), anxiety (HADS-A, *r* = 0.471), and depression (HADS-D, *r* = 0.544); however, it was not correlated with auditory thresholds (Figure 3). Multiple regression analysis identified only a score of HADS-A statistically associated with tinnitus severity (Table 4).

**Table 4:** Multiple regression analyses of tinnitus severity and the variables hearing impairment, anxiety, and depression.

	****β****
PTA on the affected side	–
SDS on the affected side	–
PTA on the contralateral side	–
SDS on the contralateral side	–
VAS	–
HADS-D	–
HADS-A	0.546^**^
	R^2^ = 0.299

We set a score of THI as a dependent variable. Also, both side of PTA and SDS, VAS, HADS-D, and HADS-A are placed as independent variables. HADS-A score as an independent predictive factor for THI. β, Standardized beta coefficient;

** p < 0.01. HADS-A, Hospital Anxiety and Depression Scale Anxiety score; HADS-D, Hospital Anxiety and Depression Scale Depression score; VAS, Visual Analog Scale for unilateral hearing impairment; PTA, pure tone audiometry; SDS, speech discrimination.

## Discussion

In the present study, we sought to determine what factors affect QoL and the clinical characteristics of tinnitus in patients undergoing conservative management of VS. Of the many subjective and audiological measurements we assessed in this prospective study, we demonstrated that tinnitus severity (THI) is the strongest predictor of QoL (SF-36) in conservatively managed VS patients. Tinnitus distress was significantly correlated with hearing impairment (VAS), anxiety (HADS-A), and depression (HADS-D). Moreover, multiple regression analysis identified HADS-A as an independent predictive factor of tinnitus severity. Patients' aging positively affected vitality and emotional and mental health aspects of QoL, and we found no relationship between QoL and facial paresis.

QoL is a primary consideration in deciding on a course of treatment for patients with VS. Active treatment with microsurgery or radiotherapy controls the tumor, but QoL could be adversely affected. With recent improvements in MR imaging and a better understanding of the characteristics of slow-growing schwannoma tumors, conservative management using a wait-and-scan protocol has provided gained popularity for VS patients. Previous analyses comparing the two treatment approaches showed that conservatively managed patients have a better QoL (33). Although we did not do a direct comparison with VS patients receiving active treatment, some symptoms of conservatively managed VS patients, such as dizziness and hearing impairment, had less impact on QoL than tinnitus did. Age of the patients also affected only vitality and emotional and mental health aspects of QoL, but not other aspects of QoL. Further research directly comparing actively and conservatively managed VS patients on multiple variables, as was done here, is required.

In the present study, the severity of audiological symptoms predominantly affected QoL. However, we observed that facial motor function did not affect QoL: this may be because more than half of our participants exhibited negligible facial paresis, as assessed by the FaCE. Only two studies have published data on audiological symptoms and QoL in patients with conservative management of VS. They found that the severity of vertigo is the strongest factor in predicting QoL. Myrseth et al. found that in patients with untreated VS, vertigo significantly correlated with SF-36 (34). It is difficult to compare our results with theirs, however, since their cohort contained participants who received active treatment, unlike any participants in our study. In a study similarly designed to ours, Lloyd et al. found that the severity of subjective symptoms, as assessed by the DHI, was the strongest predictive factor for QoL (SF-36) in conservatively managed patients (11). THI and DHI scores showed weaker, but still significant correlations with SF-36 (11). By contrast, our study indicated that tinnitus measured by THI had the strongest negative impact, and the severity of vertigo and hearing loss had weaker statistical associations with SF-36. Methodological details may account for the discrepancy between the results of previous studies and our results. Our study which showed a high response rate of 97.3% could imply likely passive participants who were elderly, apathetic, and depressed patients.

SF-36 is a well-established QoL instrument, but it is not a disease-specific instrument. Another QoL instrument is the PANQOL, which was recently developed as a disease-specific questionnaire (9). While it effectively evaluates QoL in patients with VS, it cannot assess tinnitus, as it does not contain a specific assessment item for tinnitus. This means that it cannot determine whether tinnitus distress will have a negative impact on QoL. Our results show that the assessment of tinnitus distress will help physicians understand what influences patients' QoL, especially what bothers them in their daily lives.

To the best of our knowledge, the present study is the first to analyse tinnitus distress with respect to auditory function and psychological condition of patients undergoing conservative management of VS. How might tinnitus distress relate to auditory function?

We hypothesized that tinnitus severity in VS patients managed conservatively could be affected by unilateral hearing loss, anxiety about a growing tumor and its associated symptoms, and depression related to having a chronic disorder. Generally, hearing loss is a well-known mediator of spontaneous tinnitus, associated with high level of tinnitus distress (15, 35, 36). The relationship between hearing level and tinnitus distress, however, is not straightforward. Robert indicated that many individuals who have age-related hearing loss do not show tinnitus (37). Pinto showed no statistical relationship between age, hearing threshold, and tinnitus severity (38). One study analyzed VS-related tinnitus pointed out the non-linear relationship between hearing impairment and this symptom (39). As a result in our study, VAS scores and scores on both domains of the HADS were significantly correlated with THI scores, while hearing examination failed to correlate tinnitus severity. Moreover, HADS-A was an independent predictor of tinnitus severity, as measured by THI. These findings indicate that characteristics of VS-related tinnitus mainly reflect the patients' subjective hearing handicap and psychological condition. Interestingly, a similar trend in a previous study using chronic spontaneous tinnitus population was shown that tinnitus distress significantly correlated to the degree of anxiety, while no relation to PTA (40). Because the reason behind a patient's uneasiness was individualized, it is difficult to convince how tinnitus severity generated by anxiety. Thus, it may be crucial to conducting a proper interview. From the above considerations, our results suggest that the multifactorial characteristics of VS-related tinnitus are clinically similar to primary tinnitus.

Despite the lack of a cure for tinnitus, selection of appropriate treatment will improve symptoms and alleviate distress (14, 15). As the AOHNS guideline for tinnitus strongly recommends, determining whether tinnitus is bothersome or not using a validated questionnaire will help clinicians to determine a course of treatment (14). Dobie's pyramid tinnitus concept depicted entire people who experienced chronic tinnitus: the majority of these people were not particularly bothered by this symptom (41). On the other hand, previous meta-analyses show that 70% of VS patients have subjective tinnitus (42), while only 25% of our participants had greater than mild tinnitus severity. Taken together, these results indicate a similar trend to primary tinnitus that a certain number of VS patients recognize they have tinnitus but only a proportion of them will be bothered by it.

As the primary remedy for VS-related tinnitus, education and counseling can be acceptable. In this remedy, we could explain a patient about the possible determination of pathogenesis and other treatment options. As previous studies, and our results suggest, compression of the auditory nerve by the VS (43), an abnormal firing of auditory neurons due to a lack of lateral suppression at ordinary levels of sound (15), and anxiety itself may cause patients' tinnitus. In light of the relationship between tinnitus distress and patients' psychological condition, treatment for anxiety is relevant for improving QoL. A counseling includes a cognitive approach in a broad sense. Clinical guidelines for anxiety disorders recommend cognitive behavioral therapy (44). Cognitive behavioral therapy can be effective for both tinnitus and severe anxiety disorder (14, 45), but this method requires administration by trained psychologists. For patients presenting with severe tinnitus, one should consider consulting a psychiatrist.

One possible limitation is the presence of a confounding factor. It was difficult to distinguish whether patients' psychological condition was related to VS, because the HADS is a generic questionnaire. This possible confound will be disentangled in future research. It will be useful in the future to use the PANQOL scale, since it contains an assessment for anxiety (9). Therefore, a complementary evaluation using various questionnaires will reflect the patients' QoL more precisely. Another limitation was that the number of cases in this study was relatively small; thus, our results may not be generalizable. Also, our study did not assess a long-term response to any intervention. To analyse whether an intervention adequately helps patients undergoing conservative management for VS, further data need to be collected.

In conclusion, our results suggest that characteristics of VS-related tinnitus are similar to those of primary tinnitus. Interventions designed for VS-related tinnitus should assess to what extent tinnitus bothers the patient and should reduce any unpleasant emotions that may exacerbate symptoms. This approach may improve their QoL.

## Ethics Statement

This study was approved by the ethics committee of the Keio University School of Medicine (JPRN-UMIN000008901).

## Author Contributions

TK and NO contributed to the analysis and interpretation of data, statistical analysis, and writing of the first draft. TK, TN, and NO contributed to data acquisition, interpretation of data, and review and critique of the final version of the manuscript. NO and KO was responsible for study conception and design, data acquisition, analysis and interpretation, statistical analysis, as well as reviewing and critiquing the manuscript. All authors reviewed the manuscript.

### Conflict of Interest Statement

The authors declare that the research was conducted in the absence of any commercial or financial relationships that could be construed as a potential conflict of interest.

## Abbreviations

VS: vestibular schwannoma
QoL: quality of life
THI: tinnitus handicap inventory
DHI: dizziness handicap inventory
FaCE: facial clinimetric evaluation scale
SF-36: medical outcomes study 36-item health survey short form
PF: physical functioning
RP: role physical
BP: bodily pain
GH: general health
VT: vitality
SF: social functioning
RE: role emotional
MH: mental health
PTA: pure tone audiometry
SDS: speech discrimination score
HADS-A: hospital anxiety scale
HADS-D: hospital Depression Scale
VAS: Visual Analog Scale
FDR: false discovery rate
SD: standard deviation.
